# Thanks to reviewers and to the two editors emeriti

**DOI:** 10.1057/s41271-020-00227-0

**Published:** 2020-05-04

**Authors:** Elena N. Naumova

**Affiliations:** Division of the Nutrition Epidemiology and Data Science, Friedman School of Nutrition Science and Policy, Tufts University, Boston, USA

Following the established tradition, each year we recognize the invaluable contribution of our reviewers to the *Journal of Public Health Policy (JPHP)*. You will see this below.

As part of my first editorial as the Editor-in-Chief, I would also like to use this opportunity to recognize Dr. Phyllis Freeman and Dr. Anthony Robbins, now Editors Emeriti, for their outstanding dedication to the *Journal* for over 16 years.

Since 2004, when the *Journal* moved to the Palgrave Macmillan web-based submission and review system and to their contractor, EJPress of Bethesda, MD, USA, the *Journal* has grown so much in its reach to international audiences. Such success can be attributed to a strong partnership between the *JPHP* and the World Federation of Public Health Associations (WFPHA) that was proposed by Tony and skillfully nurtured by Phyllis in her meticulous editing and frequent trips abroad. The establishment of this partnership was a pivotal moment for both institutions. It marked for the *JPHP* the establishment of a sought-after link with the global community of public health practitioners, scholars, and researchers. For the WFPHA, at that time led by Dr. Ulrich Laaser, it represented a much-needed opportunity to increase the Federation’s visibility and a means for its member public health associations to communicate their activities and achievements to a global audience. It built upon Phyllis and Tony’s desire to “close the publishing gap” by offering a means for the members of public health communities “closest to poverty and poor health in the developing world” [1] to have access to and facilitate public health science and practice communication within an international peer-reviewed journal. Most importantly, Tony and Phyllis had created an international community of passionate thinkers. Here are just a few testimonials from the *Journal*’s loyal supporters:

The world is a better place because of Tony and Phyllis’s deep commitment to public health. *JPHP* is one major way they have made a difference, though their care, skill, expertise, and exquisite discernment exemplified by their meticulous editing.

David Ozonoff, MD, MPH

Over the years - no, decades - they [Tony and Phyllis] have shown themselves not only to be extremely dedicated and skilled editors but also real comrades in a mutual effort to make this world a better place. Through their stewardship of the *Journal*, during some really difficult periods, they have kept together one of the few places where concerns about social justice, scholarship, and academic integrity are truly one. All too many journal editors have forgotten that public health was once not only an exercise in professional and technical competence but also a ‘calling,’ a place where skills and training could be used to build a better, more just, world.

David Rosner, Ph.D.

Since first meeting Phyllis and Tony at the World Congress on Public Health in 2004, I’ve watched and applauded their tireless efforts to make the *JPHP* a ‘go-to’ publication for the global public health community. I’ve admired their high-quality editorial guidance on the content of *The Federation’s Pages* that appear in each issue of the *JPHP*. They consistently provided wise counsel to the WFPHA as it charted its path through the crowded world of global health, including during my two-year term (2012-2014) as the WFPHA’s President. They did so with grace, whenever asked, and always with a lovely, calm smile on their faces. They made a significant contribution to the global public health community, one that will have positive impacts for years to come.

James Chauvin, MA, MSc, HonFFPH

[Tony and Phyllis] approach public health not as a discipline, but as drawing on the best evidence across disciplines to solve public health challenges. They embodied this approach through their expertise and experience in medicine, epidemiology, law, politics and policy, teaching, writing, and activism. They saw *JPHP* as a tool in creating communities of concerned public health practitioners and researchers around the world, including through WFPHA, and the thrill of making connections with such *joie de vivre*. Their dedication and personal attention to building the skills and capacities of authors in joining the *JPHP* community are also remarkable. Through all their work the spirit of social justice shone through, and the commitment to speak truth to power, and to use knowledge as a compass to direct our collective way forward to a healthier more equitable world.

Shyama Kuruvilla, Ph.D.

I had the honor to work with Tony and Phyllis and witness the tireless efforts they put into promoting global public health, through supporting authors from resource-limited settings, clarifying fundamental issues in public health, pushing for critical public health priorities such as vaccine accessibility, mentorship and many other endeavors. Their dedication to global population health has been deeply felt in our exchanges and throughout the course of collaborations. Their vision, passion and drive have made *JPHP *such a unique jewel in public health literature.

Julia Wu, Sc.D.

***

## Thanks to reviewers

**Fig. 1:**
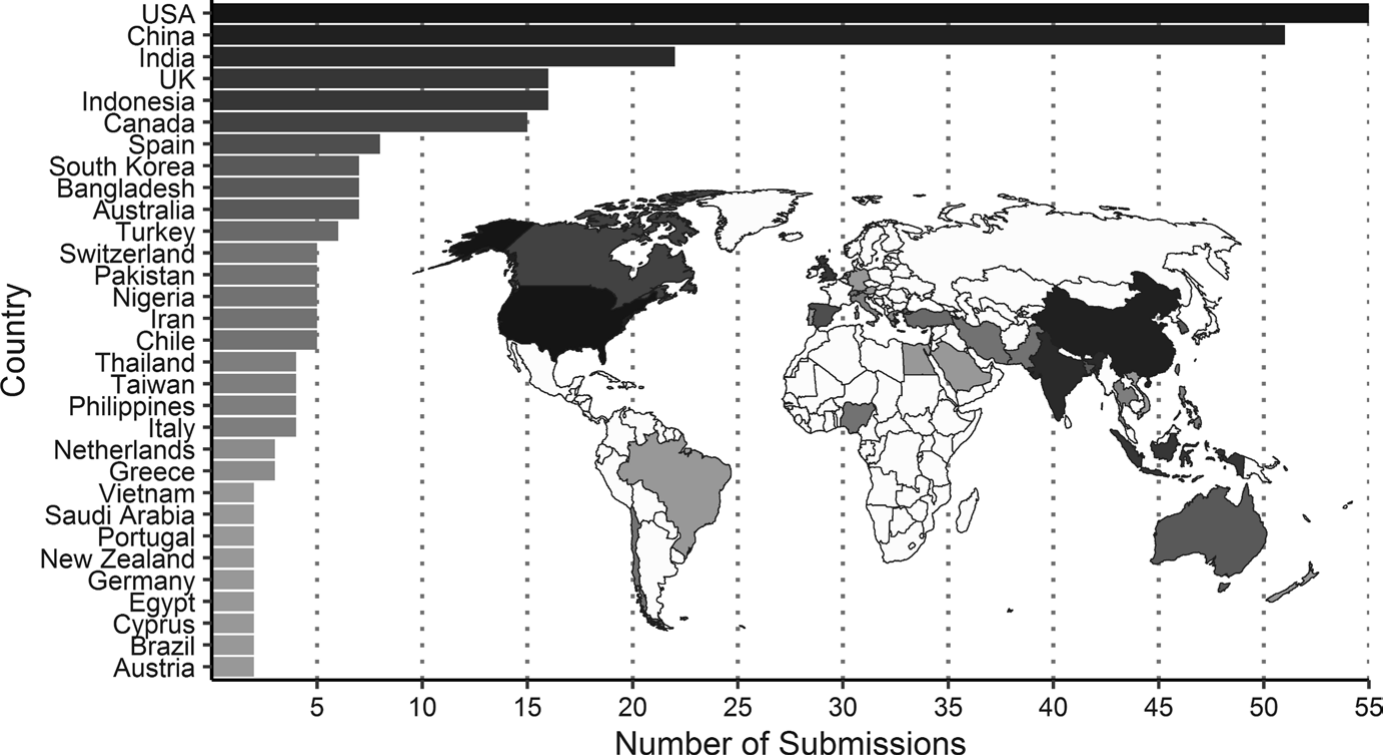
*JPHP* received two or more manuscripts from 31 countries in 2019

In 2019, we received 465 submissions from more than 43 countries, with more than ten submissions from the United States of America, China, India, the United Kingdom, Indonesia, and Canada (Fig. 1). Authors from Argentina, Australia, Austria, Bangladesh, Belgium, Brazil, Canada, Chile, Cyprus, Denmark, Egypt, Ethiopia, French Guiana, Germany, Greece, Hong Kong, Iran, Ireland, Israel, Japan, Korea (south), Republic Of Lebanon, Mexico, Morocco, Nepal, Netherlands, New Zealand, Oman, Pakistan, Peru, Philippines, Russia, Saudi Arabia, South Africa, Spain, Switzerland, Taiwan, Thailand, Trinidad And Tobago, Turkey, United Arab Emirates, Uruguay, and Vietnam also contributed manuscripts.

As I have learned from Phyllis and Tony, one of the most challenging tasks is to find talented reviewers who give us advice and help the authors of promising submissions to make them better. We thank the constructive guidance and timeliness provided by 81 reviewers who completed 106 reviews over the past year. In particular, we thank our champions who crafted multiple reviews: Roy Widdus (10) and Richard Daynard (5). We would like to express our gratitude to:

**Table:** 

Folashade Agusto	Kenneth Kalu	Ambele Judith Mwamelo
Miriam Alvarado	Ben Kelley	Carles Muntaner
Manal Al Halabi	Alexandra Kulinkina	Marion Nestle
John Balbus	Philip Landrigan	Benjamin Oosterhoff
Arindam Basu	Miriam Laugesen	Blake Poland
Mercedes Becerra	DeAnn Lazovich	Jennifer Pomeranz
Yonatan Ben-Shalom	Ruth Levine	Jirair Ratevosian
Ruth Berkelman	John Glasser	David Roodman
William Bor	Lisa Gualtieri	Laetitia Rispel
Donald Bundy	Kenneth Hartigan-Go	Hanna-Andrea Rother
Richard Clapp	Stephane Horel	Najat Saliba
Enrique Cifuentes	Charles Johnson	Joshua Sharfstein
Jennifer Coates	Mahasti Kahkpour	Bruce Snyder
Ryan Cramer	Jennifer Kates	Sathyanarayana Tamysetty
Adolfo Cuevas	Karen Kosinski	Michael Touchton
Mags Currie	Joseph Kyebuzibwa	Duong Tran
Jonathan Darrow	Heidi Larson	Cathy Walker
Richard Daynard	Hannah Lawman	Jim te Water Naude
Faran Emmanuel	Richard Lemen	Chen Wei
Gary Franklin	Martha Livingston	Wylin Wilson
Francesco Fusco	Madhumita Dobe	Karen van Unen
Omid Gilanpour	Naomi Mann	Julie Wang
Mark Gottlieb	Adolfo Martinez Valle	Roy Widdus
Razsak Gyasi	Chris Mason	Sergut Wolde-Yohannes
Carolyn Heckman	Sonia Menon	Mahrukh Yousaf
Dagmawi Iyasu	Karen Milton	Friedo Zölzer
Timothy Jones	Elias Michaelides	Guus Zwitser

## The path ahead

This year the *Journal* is transitioning to a new manuscript management system supported by Springer Nature Publishing. This system offers better functionality, tracking, and sharing the progress with our readers, reviewers, and the members of Editorial Board.

As a reader of *JPHP*, I enjoyed the breadth and quality of material the previous editors selected and pursued, especially the thought-provoking papers accompanied with commentaries. As a teacher, I know that in *JPHP* I always am able to find an exciting communication for my students to spark a heated discussion. As a reviewer and editor, I enjoyed working with authors who bring new ideas, wicked problems, and timely solutions. I especially treasure my experience working with the diverse audience. It is fascinating to see how a locally driven question can gain a global perspective, how an emerging global trend is affecting regional health strategies and interventions, and how authors are bringing their own perspectives through stories valuable to so many. Moving forward together with the broad network of the *Journal*’s followers and our knowledgeable and enthused Editorial Board, we will strive to keep *JPHP*’s strong traditions of inclusiveness, global outlook, and readiness to tackle difficult issues.

The *Journal* is well positioned to continue its well-established traditions as a publication with a progressive perspective on population health. In the era of artificial intelligence and big data, deep learning and automation, global surveillance and early warning systems, the health strategies, policies, and interventions aiming to protect health, prevent injury and illness and promote health at the population, community, and environmental levels will only gain importance and relevance. I have no doubt that both cutting-edge academic research topics and routine challenges of health practitioners will have no shortage of thought-provoking and controversial issues.
